# Zero Healthcare-Associated COVID-19

**DOI:** 10.1017/ash.2021.142

**Published:** 2021-07-29

**Authors:** Moi Lin Ling, Molly How, Kwee Yuen Tan, Elaine Wee, Phoon Poh Choo, Lai Chee Lee

## Abstract

**Background:** The ongoing COVID-19 pandemic tests the healthcare system in many ways. The scarcity of resources poses challenges to infection prevention (IP) practices. We describe our experience in managing such scarcity in our care of COVID-19 patients in the hospital as well as community settings. **Methods:** The hospital pandemic plan traditionally included only plans for healthcare delivery management within the hospital. However, on March 25, 2020, a decision was made by the Ministry of Health to set up swab isolation (SIFs) and community care facilities (CCFs) to meet the growing demand for isolation beds for migrant workers infected by COVID-19. The CCFs were located in convention halls and resort centers and the SIFs were located in facilities previously functioning as hotels. Mobile medical teams were activated to run clinics at the dormitories housing 200,000 migrant workers. The IP team of an acute- and tertiary-care hospital in Singapore was activated to oversee IP measures at facilities managed by medical teams from the hospital, with the goal of zero healthcare-associated COVID-19 cases among staff. Two IP leaders were set up to oversee the IP program at 8 dormitories, 4 SIFs, and 2 CCFs. In total, 12 IP staff and 15 infection prevention liaison officers (IPLOs) were deployed from 2 acute-care hospitals and 3 specialty centers to conduct training in hand hygiene and the use of personal protective equipment, and to conduct daily audits of compliance to practice guidelines. Education on personal hygiene was also given to patients in these facilities in at least 7 languages. In the SIFs and dormitories, IPLOs were recruited to perform daily audits and feedback to the IP team on issues related to IP at the sites. **Results:** Since our first COVID-19 patient on January 23, 2020, there has been no report of healthcare-associated COVID-19 within the hospital nor among the medical, administrative, and support service staff working in the external operation facilities. Daily audits showed an average of 99.4% compliance to IP guidelines. **Conclusions:** IPLOs or IP champions play a significant role in ensuring compliance to IP guidelines. This compliance allows the IP professional to focus on the evaluation of the IP program, managing IP consultations, and planning and implementation of the IP program in nontraditional healthcare settings. The key success factors of the program included the ability to contextualize the planning and implementation of IP programs in various settings, strong leadership support, cohesive teamwork, and effective communication at various levels.

**Funding:** No

**Disclosures:** None

